# Multivariate Statistical Evaluation of Ginseng Quality and Antioxidant Activity

**DOI:** 10.3390/molecules31162932

**Published:** 2026-08-21

**Authors:** Menglin Yuan, Feilong Yang, Jie Li, Haidan Zou, Yang Liu, Heyuan Jiang

**Affiliations:** 1 College of Food and Biological Engineering, Chengdu University, Chengdu 610106, China; 17208289503@163.com (M.Y.); shuimu119@126.com (Y.L.); 2 Institute of Urban Agriculture, Chinese Academy of Agricultural Sciences, Chengdu 610213, China; yangfeilong@caas.cn; 3 Plant Resource Team, National Chengdu Agricultural Science and Technology Center, Chengdu 610213, China; jieli0525@163.com (J.L.); zellazou@foxmail.com (H.Z.)

**Keywords:** ginsenosides, antioxidant activity, correlation, multivariate statistical analysis

## Abstract

The composition of ginsenosides is closely associated with antioxidant activity, but the effects of variety and growth year on the accumulation of individual ginsenosides and their quantitative relationships with free radical scavenging activity remain unclear. In this study, six ginsenosides (Rb1, Rb2, Rc, Rd, Re, and Rg1) in cultivated ginseng (*Panax ginseng* C. A. Mey.) and American ginseng (*Panax quinquefolius* L.) were quantified by HPLC, combined with DPPH and ABTS assays, and systematically analyzed using PCA, OPLS-DA, HCA, and PLSR. The results showed that variety exerted a much greater influence than growth year. American ginseng contained significantly higher levels of PPD-type ginsenosides (Rb1, Rd, Rc), while Rg1 was more abundant in cultivated ginseng. OPLS-DA clearly distinguished the two varieties based on ginsenoside profiles, with Rc, Rb1, and Rd identified as key marker ginsenosides (VIP > 1). Ginsenoside extracts showed scavenging abilities against both DPPH and ABTS. However, the PLSR model only successfully yielded predictive capability for DPPH. The poor prediction for ABTS suggested that ABTS scavenging may be attributed to other untested constituents in the extracts. This study provides a robust basis for variety-oriented quality control and antioxidant efficacy evaluation in ginseng products.

## 1. Introduction

Ginseng (*Panax ginseng* C. A. Mey.) is a traditional and valuable Chinese medicinal herb, known for its effects in relieving collapse, improving intelligence and calming the mind, and alleviating fatigue. It is widely used in pharmaceuticals, health products, and functional foods [1,2]. American ginseng (*Panax quinquefolius* L.), as a closely related species within the same genus, is also extensively cultivated and utilized [3]. Modern pharmacological studies indicate that ginsenosides are the most important active constituents of *Panax* species. Based on their aglycone structures, they can be classified into protopanaxadiol-type (PPD, e.g., Rb1, Rb2, Rc, Rd) and protopanaxatriol-type (PPT, e.g., Re, Rg1). Different ginsenoside monomers exhibit distinct biological activities; for example, PPD-type ginsenosides are prominent in neuroprotection and anti-apoptosis, whereas PPT-type ginsenosides are more associated with anti-inflammatory effects and improvement of learning and memory [4,5,6].

The antioxidant effect of ginsenosides is one of the foundations of their diverse pharmacological actions. Oxidative stress is closely linked to aging, cardiovascular diseases, neurodegenerative disorders, and other conditions. Ginsenosides can exert antioxidant effects through direct free radical scavenging, enhancing the activities of superoxide dismutase (SOD) and glutathione peroxidase (GSH-Px), and activating the Nrf2/ARE signaling pathway, among other mechanisms. However, most current studies focus on total saponins or only a few monomers, lacking systematic evaluation of the synergistic or antagonistic effects of different monomer combinations and their selective scavenging capacities toward different types of free radicals. Among the commonly used in vitro antioxidant assays, the DPPH method evaluates lipophilic radical scavenging activity, while the ABTS method evaluates hydrophilic radical scavenging activity. The combination of these two assays provides a more comprehensive reflection of the antioxidant profile of samples [7].

In terms of ginseng quality evaluation, traditional standards primarily rely on appearance traits, growth years, and total saponin content, with the general belief that “longer growth years and higher grades indicate superior ginseng quality.” However, increasing studies have challenged this view in recent years. Some researchers have found that the contents of certain ginsenoside monomers do not continuously increase with growth years and may even decline [8,9,10]. In addition, cultivated ginseng and American ginseng exhibit significant differences in ginsenoside accumulation patterns, which may be related to genetic background, cultivation environment, and differential expression of key metabolic enzymes such as glycosyltransferases and cytochrome P450. A few studies have observed negative correlations between PPD- and PPT-type ginsenosides, suggesting that the two types may compete for common precursor substrates [11]. More notably, Rg1 and Re, both belonging to the PPT type, also show negative correlations in some samples, indicating that downstream glycosylation branches may involve more refined flux regulation [10,12,13]. However, these metabolic associations are mostly based on visual observations and lack validation through multivariate statistical methods.

In recent years, multivariate statistical methods such as principal component analysis (PCA), orthogonal partial least squares-discriminant analysis (OPLS-DA), hierarchical clustering analysis (HCA), and partial least squares regression (PLSR) have been widely applied in the identification, active ingredient screening, and quality evaluation of Chinese medicinal materials [14,15,16,17]. These methods can effectively handle high-dimensional data and reveal latent relationships and predictive capabilities among variables. For example, PLSR can establish quantitative structure–activity relationships between ginsenoside contents and antioxidant activities, identifying key active components. However, to date, studies that simultaneously incorporate variety, grade, ginsenoside monomer profiles, and dual free radical scavenging capacities into a unified analytical framework remain scarce, and quantitative elucidation of the metabolic competition among ginsenoside monomers and its contribution to selective antioxidant effects is still insufficient.

Therefore, this study focused on cultivated ginseng and American ginseng of different grades (corresponding to growth years). Ginsenosides were extracted using ultrasound-assisted dual-enzyme extraction, and the contents of six major monomeric ginsenosides (Rb1, Rb2, Rc, Rd, Re, and Rg1) were quantified by HPLC. DPPH and ABTS free radical scavenging activities were also determined. Through multivariate statistical methods including PCA, OPLS-DA, HCA, Pearson correlation analysis, and PLSR, we systematically investigated the effects of variety and growth year on ginsenoside accumulation patterns, metabolic associations among ginsenoside monomers, and their quantitative relationships with scavenging activities against different types of free radicals. The results will provide a scientific basis for ginseng variety identification, quality evaluation, and screening of antioxidant active components, and offer clues for deeper understanding of the metabolic regulatory network of ginsenosides and their selective radical-scavenging mechanisms.

## 2. Results and Discussion

### 2.1. Comparison of Ginsenoside Contents Among Different Varieties and Grades

**Figure 1:**
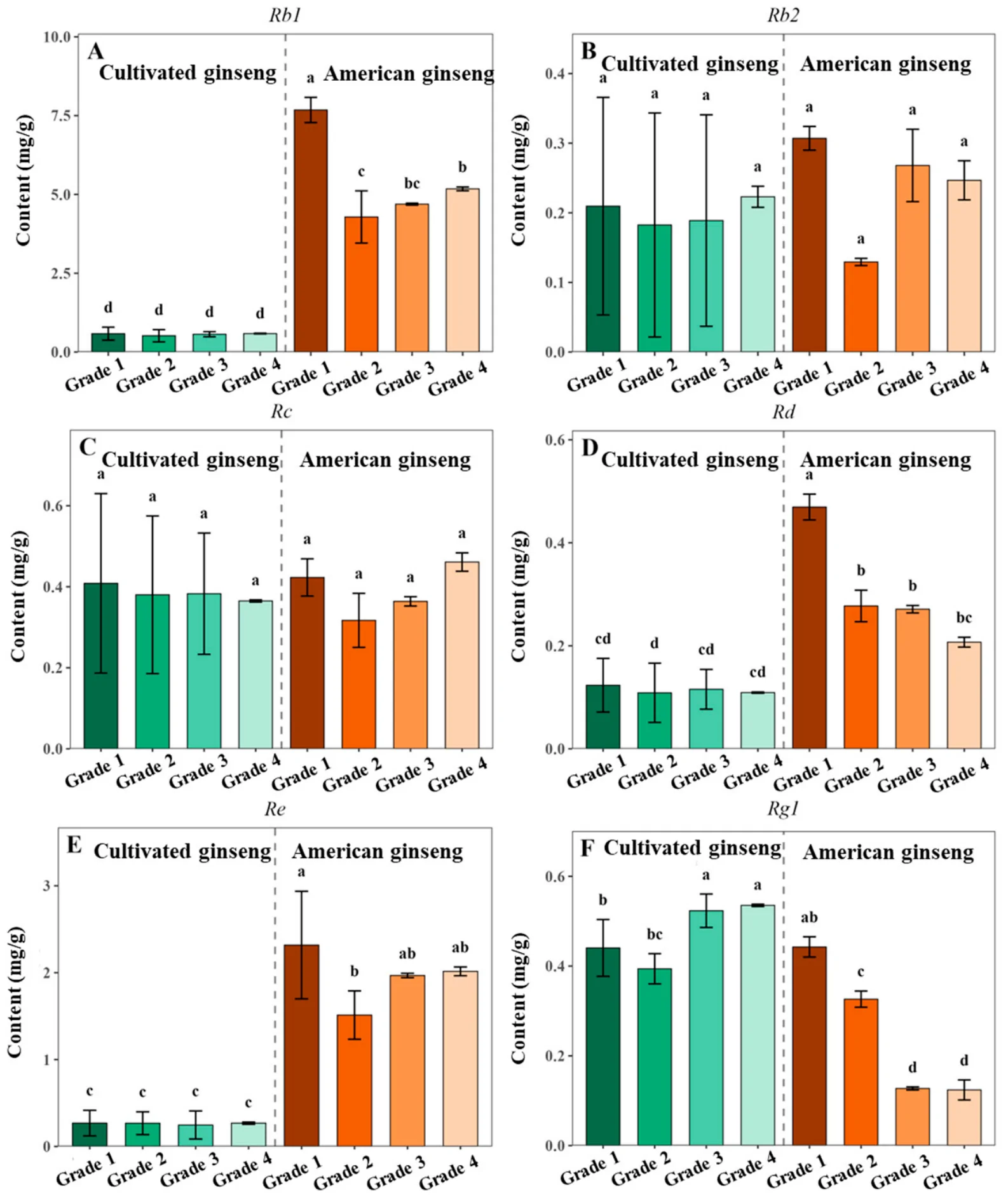
Comparison of six ginsenoside monomer contents in different varieties (cultivated ginseng and American ginseng) and grades (grades 1–4): (**A**) Rb1; (**B**) Rb2; (**C**) Rd; (**D**) Rc; (**E**) Re; (**F**) Rg1. The lowercase letters on the bar graph represent the significant differences in ginsenoside content among different ginseng varieties and grades.

By comparing the contents of six ginsenoside monomers (Rb1, Rb2, Rc, Rd, Re, Rg1) in cultivated ginseng and American ginseng across four grades (Figure 1), we found that the contents of Rb1, Rd, and Rc in American ginseng were significantly higher than those in cultivated ginseng (*p* < 0.05), while Rg1 was more abundant in cultivated ginseng. This difference may be related to the genetic background of the two ginseng types and differential expression of key enzymes in saponin biosynthesis, such as glycosyltransferases and cytochrome P450. Previous studies have reported a stronger tendency for PPD-type ginsenoside (e.g., Rb1) accumulation in American ginseng [10,12]. No significant differences were observed for Rb2 and Re between the two varieties, suggesting that the metabolic regulation of these two ginsenosides is relatively conserved across varieties.

The effect of different grades (reflecting growth years) on ginsenoside contents did not show a consistent pattern. In cultivated ginseng, higher grades (grade 1 and grade 2) did not show significantly higher contents than lower grades (grade 3 and grade 4); in fact, Rg1 was even higher in lower grades, possibly related to conversion or degradation of Rg1 to other ginsenosides in later growth stages. In American ginseng, Rd and Rg1 showed a certain positive correlation with grade, and grade 1 American ginseng had significantly higher Rb1 than other grades, indicating that ginsenoside accumulation in American ginseng may be more dependent on growth years. Overall, the effect of variety on the ginsenoside profile was far greater than that of grade, and the traditional view that “longer growth years yield richer components” needs to be re-examined at the monomeric ginsenoside level.

### 2.2. Comparison of Antioxidant Activity Among Different Varieties and Grades

**Figure 2:**
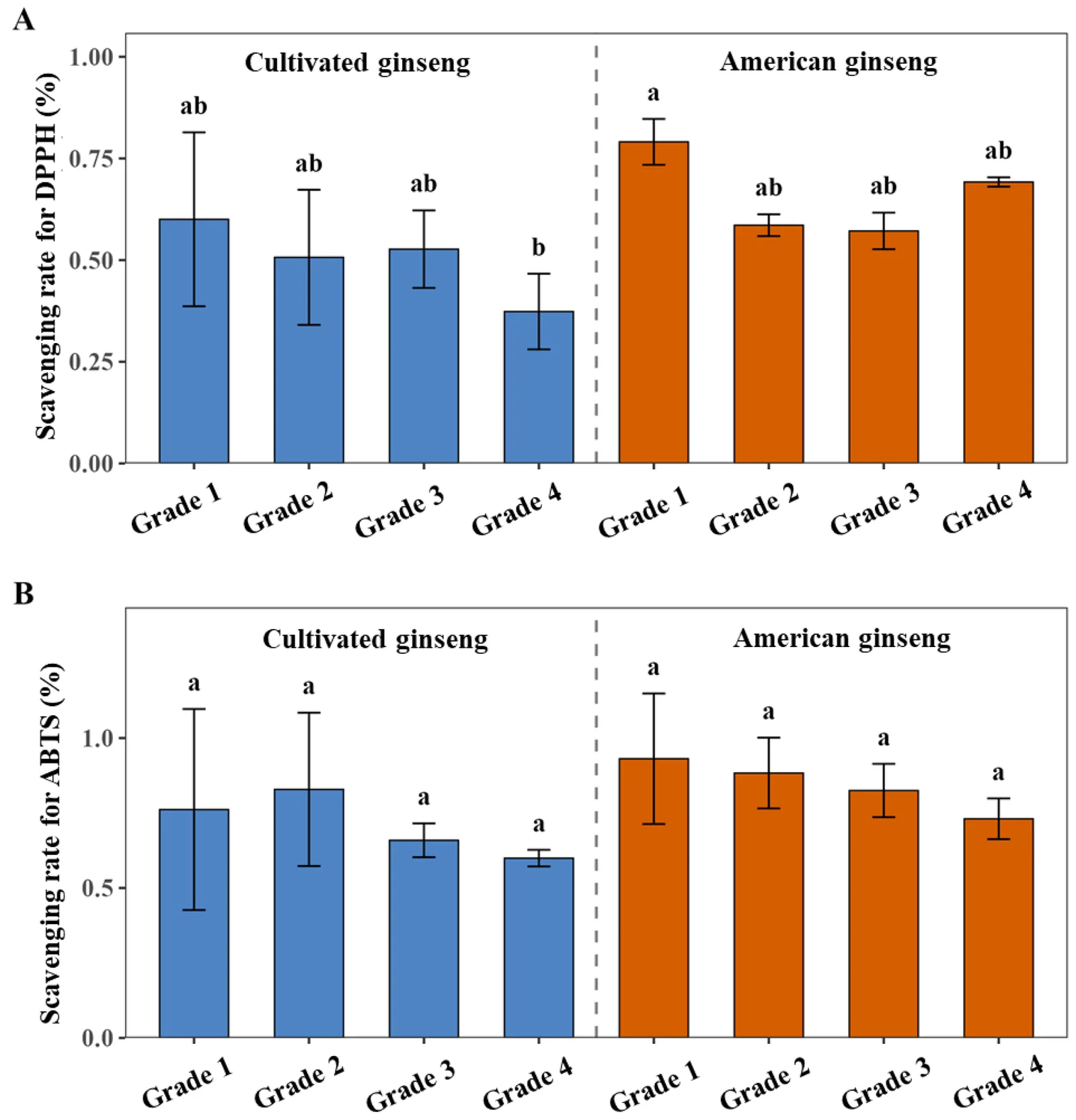
Comparison of free radical scavenging activities of different varieties and grades of ginseng samples: (**A**) DPPH scavenging rate; (**B**) ABTS scavenging rate. The lowercase letters on the bar graph represent the significant differences in antioxidant activities among different ginseng varieties and grades.

The DPPH and ABTS scavenging assays showed that both cultivated ginseng and American ginseng possessed strong antioxidant activity (scavenging rate > 50%). The antioxidant activity of American ginseng was slightly higher than that of cultivated ginseng, but the difference was not significant (Figure 2). This trend is consistent with the higher contents of PPD-type ginsenosides (e.g., Rb1, Rd) in American ginseng. PPD-type ginsenosides, with their hydrophobic skeletons, are more likely to embed into lipid environments and may have stronger scavenging capacity against lipophilic radicals such as DPPH. However, no significant effect of grade on antioxidant activity was observed under either assay, suggesting that antioxidant activity may be influenced by synergistic effects of multiple ginsenosides and that grade alone is insufficient to effectively predict antioxidant capacity [18,19].

### 2.3. Factorial Analysis of Variance for Variety and Grade Effects

To quantitatively evaluate the effects of variety, grade, and their interaction, a two-way ANOVA was performed on the balanced dataset (2 varieties × 4 grades × 3 replicates), with the results presented in Table 1. The results showed that variety significantly affected all six ginsenoside monomers (*p* < 0.001 for each), whereas grade also exerted significant effects on five of the six monomers (*p* < 0.001 for all except Re, *p* = 0.065). Notably, the F-values for variety were substantially larger than those for grade (e.g., 1287.65 vs. 35.06 for Rb1), indicating that variety was the dominant factor shaping the ginsenoside profile. Significant variety-by-grade interactions were observed for Rb1, Rb2, Rc, Rd, and Rg1 (*p* < 0.001 for all), indicating that the grade-dependent accumulation of these ginsenosides was variety-specific. For instance, Rb1 changed markedly with grade in American ginseng but remained relatively stable in cultivated ginseng (interaction *p* = 7.56 × 10^−7^).

Regarding antioxidant activity, DPPH scavenging capacity was significantly affected by grade (*p* < 0.001) and showed a significant variety-by-grade interaction (*p* = 0.002), whereas the variety main effect was not significant (*p* = 0.079). For ABTS, grade also showed a significant main effect (*p* = 0.003), while neither variety nor the interaction reached significance (*p* = 0.639 and 0.151, respectively). The contrasting interaction patterns indicate that the effect of growth year on DPPH scavenging activity is variety-dependent, whereas its effect on ABTS scavenging activity is consistent across varieties.

### 2.4. Multivariate Pattern Recognition Analysis

#### 2.4.1. Variety Discrimination Based on Ginsenoside Profiles

**Figure 3:**
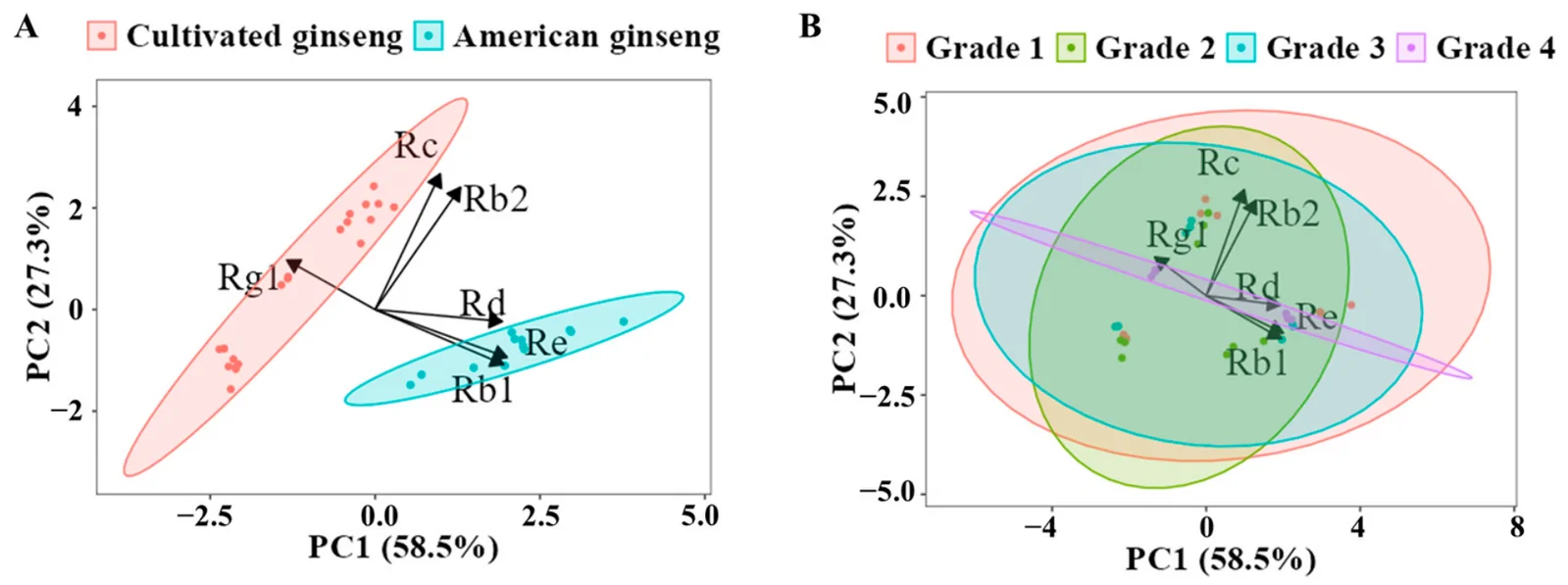
Principal component analysis (PCA) score plots based on six ginsenoside contents: (**A**) labeled by variety (cultivated ginseng vs. American ginseng); (**B**) labeled by grade (grades 1–4).

The PCA loading plot (Figure 3A) showed that Rg1 was negatively correlated with Rb1, Re, and Rd. Notably, Rg1 and Re, both belonging to the PPT-type ginsenosides, also exhibited a negative correlation. This observation suggests that the accumulation patterns of ginsenosides, governed by the underlying metabolic regulation, may not be simply divided into PPD/PPT types, but rather involve more refined downstream branch-pathway competition [20,21]. However, further elucidation of branch-pathway regulation in ginsenoside metabolism requires robust molecular-level evidence.

**Figure 4:**
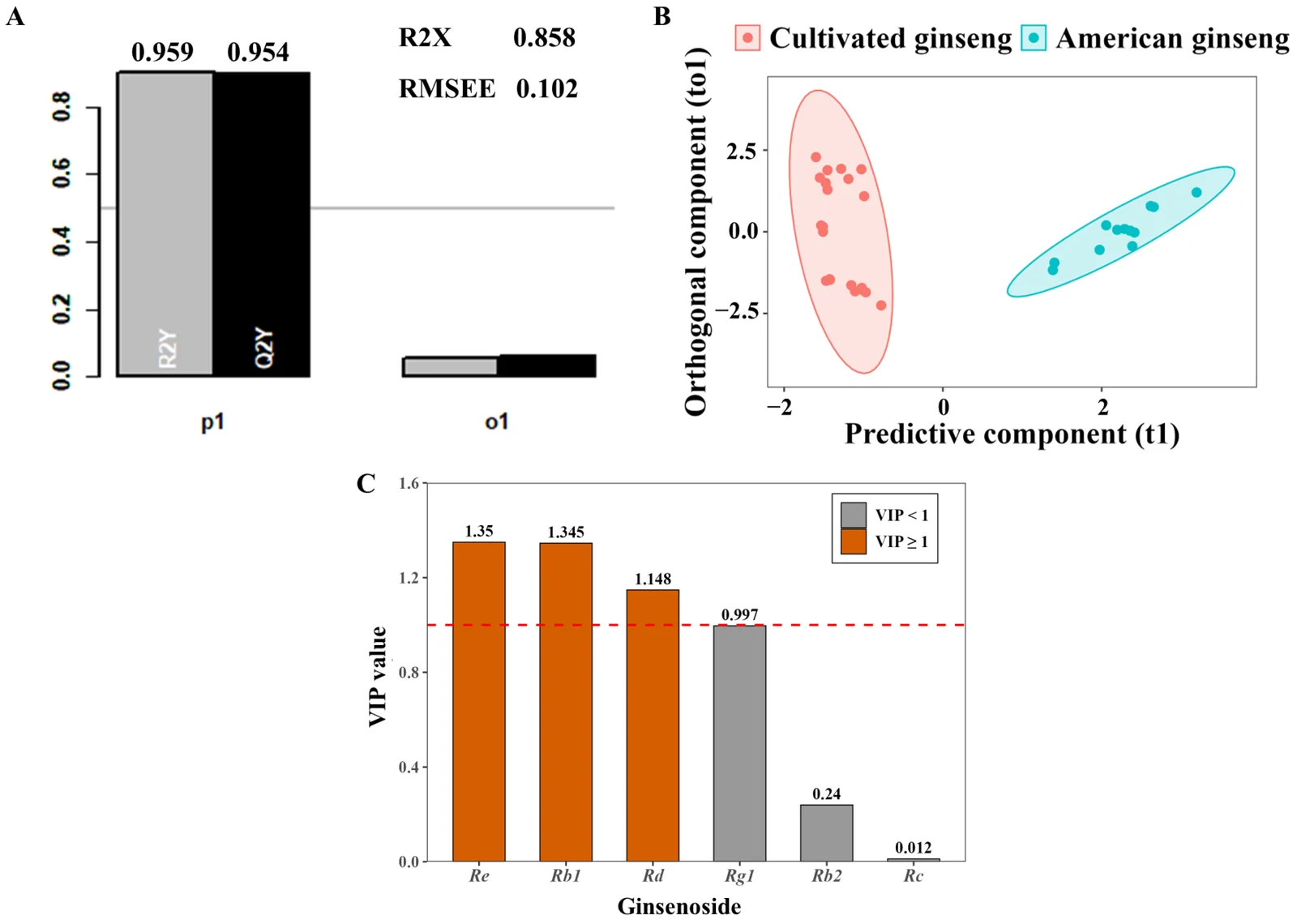
Orthogonal partial least squares-discriminant analysis (OPLS-DA) results based on six ginsenoside contents: (**A**) validation plot; (**B**) score plot; (**C**) variable importance in projection (VIP) plot. Horizontal reference lines: the gray in (**A**) indicates the cutoff for goodness of fit and predictive validity; the red dashed in (**C**) indicates the VIP threshold for variable importance.

In the PCA plots, samples clustered clearly by variety but showed no separation by grade (Figure 3A,B), reinforcing that variety has a greater influence on the ginsenoside profile than grade. The OPLS-DA model, successfully differentiating the two varieties of ginseng, showed good goodness-of-fit ($R_{X}^{2}$ = 0.858, $R_{Y}^{2}$ = 0.959) and predictive ability ($Q_{Y}^{2}$ = 0.954) (Figure 4A,B). Permutation tests (200 iterations) confirmed the validity of the model, with a negative Q_2_ intercept (–0.258) and significant *p* values ($p_{R_{Y}^{2}}$ = 0.005, $p_{Q^{2}}$ = 0.005), indicating that the model was not overfitted and the observed discrimination between varieties was highly significant. The key ginsenosides with VIP values > 1 were Rc, Rb1, and Rd (Figure 4C), indicating that they are the signature components for distinguishing cultivated ginseng from American ginseng.

#### 2.4.2. Hierarchical Clustering Analysis (HCA) of Ginsenoside Profiles

**Figure 5:**
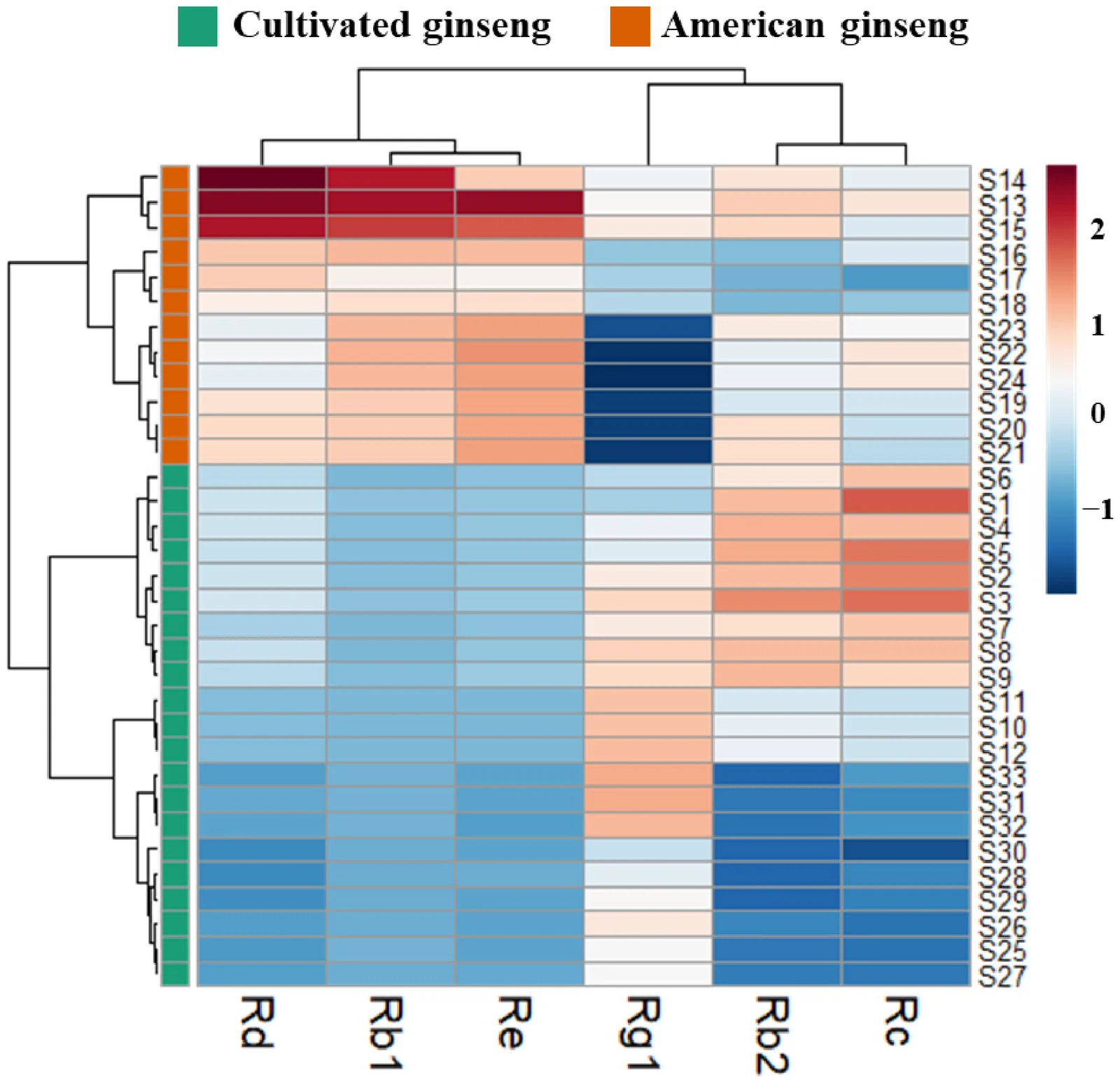
Hierarchical clustering analysis (HCA) dendrogram based on six ginsenoside contents.

As shown in Figure 5, cultivated ginseng and American ginseng were divided into two major clusters. Meanwhile, the ginsenoside clustering showed that Rd, Rb1, and Re formed one group; Rg1 stood alone; and Rb2 and Rc formed another group, indicating that the accumulation patterns of these ginsenosides were differentially regulated across the samples. Notably, Rb1 and Re were closest in the dendrogram, suggesting that their abundance variations were most similar, despite belonging to different aglycone types (PPD and PPT, respectively). This clustering result may serve as a reference for prioritizing candidate ginsenoside pairs for further pathway correlation analysis.

### 2.5. Correlation Analysis Between Antioxidant Activity and Ginsenoside Contents in Different Origins

#### 2.5.1. Pearson Correlation Analysis

**Figure 6:**
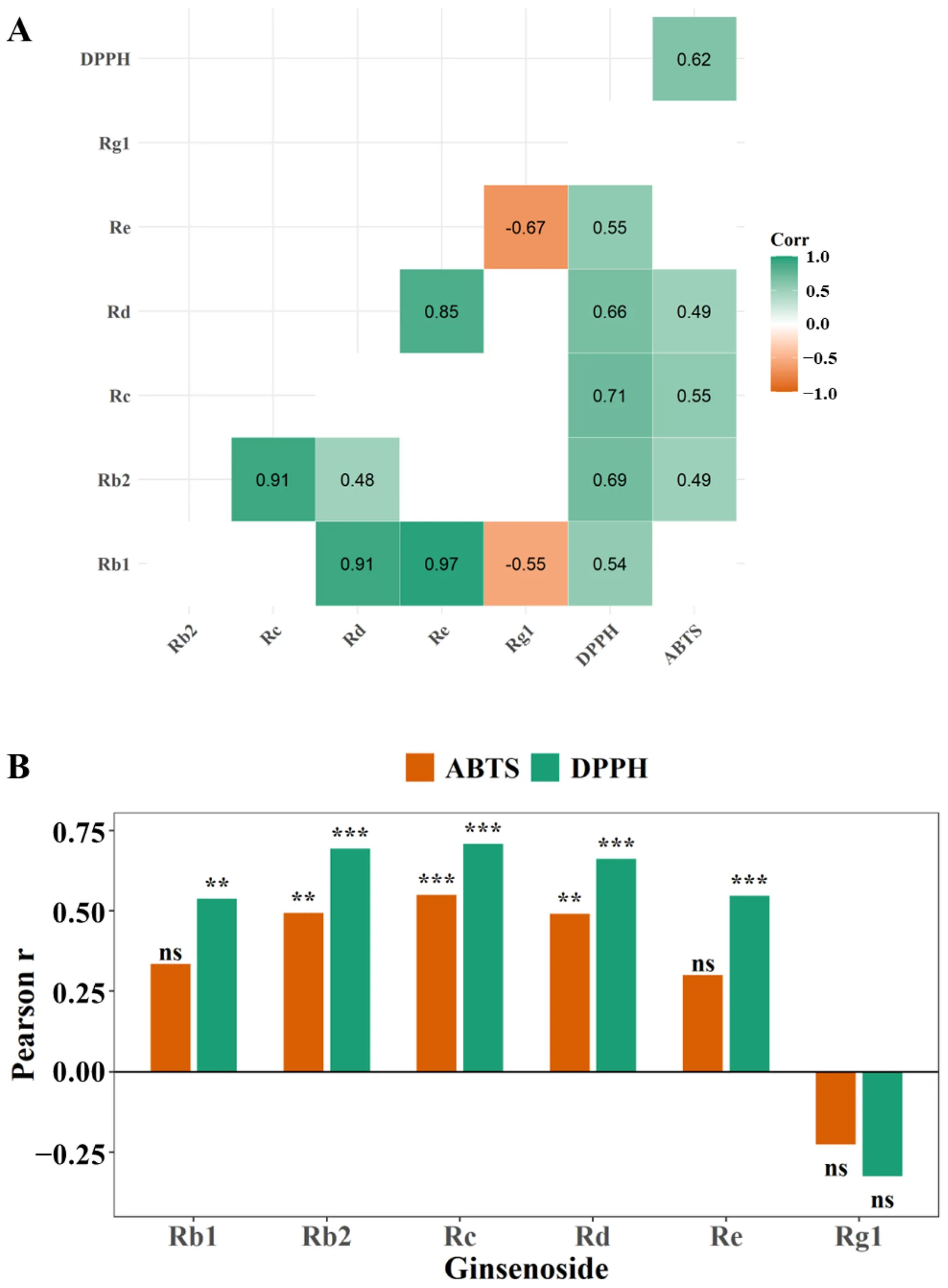
Pearson correlation analysis of ginsenoside monomer contents with DPPH and ABTS scavenging activities: (**A**) correlation heatmap; (**B**) significance test (*p*-values). Asterisks indicate significance levels (**: *p* < 0.01; ***: *p* < 0.001), and “ns” denotes not significant (*p* ≥ 0.05). Color intensity and asterisks represent the strength and significance of Pearson correlation coefficients.

Pearson correlation analysis (Figure 6) showed that DPPH scavenging rate was positively correlated with all ginsenoside monomers except Rg1, with stronger correlations observed for Rb2, Rd, and Rc (r > 0.5). ABTS scavenging rate was significantly correlated only with Rd, Rc, and Rb2 (r > 0.5), while no significant correlation was found with Rb1, Re, or Rg1. This difference may be related to the nature of the free radicals: DPPH is a lipophilic radical, and the hydrophobic moieties of PPD-type ginsenosides (e.g., Rb1, Rd, Rc, Rb2) are more likely to interact with it; ABTS is a hydrophilic radical, requiring more hydrophilic molecular structures, while Rd, Rc, and Rb2 may possess an appropriate hydrophilic–lipophilic balance. Although Rg1 is a PPT-type with better water solubility, its antioxidant mechanism may not depend on direct radical scavenging, or it may require synergy with other components. However, given the substantial collinearity among the PPD-type ginsenosides, these pairwise correlation coefficients should be interpreted with caution, and multivariate regression models were subsequently employed to disentangle the net contributions.

#### 2.5.2. Quantitative Structure–Activity Relationship of Ginsenosides–Antioxidant Activity Based on Partial Least Squares Regression (PLSR)

**Figure 7:**
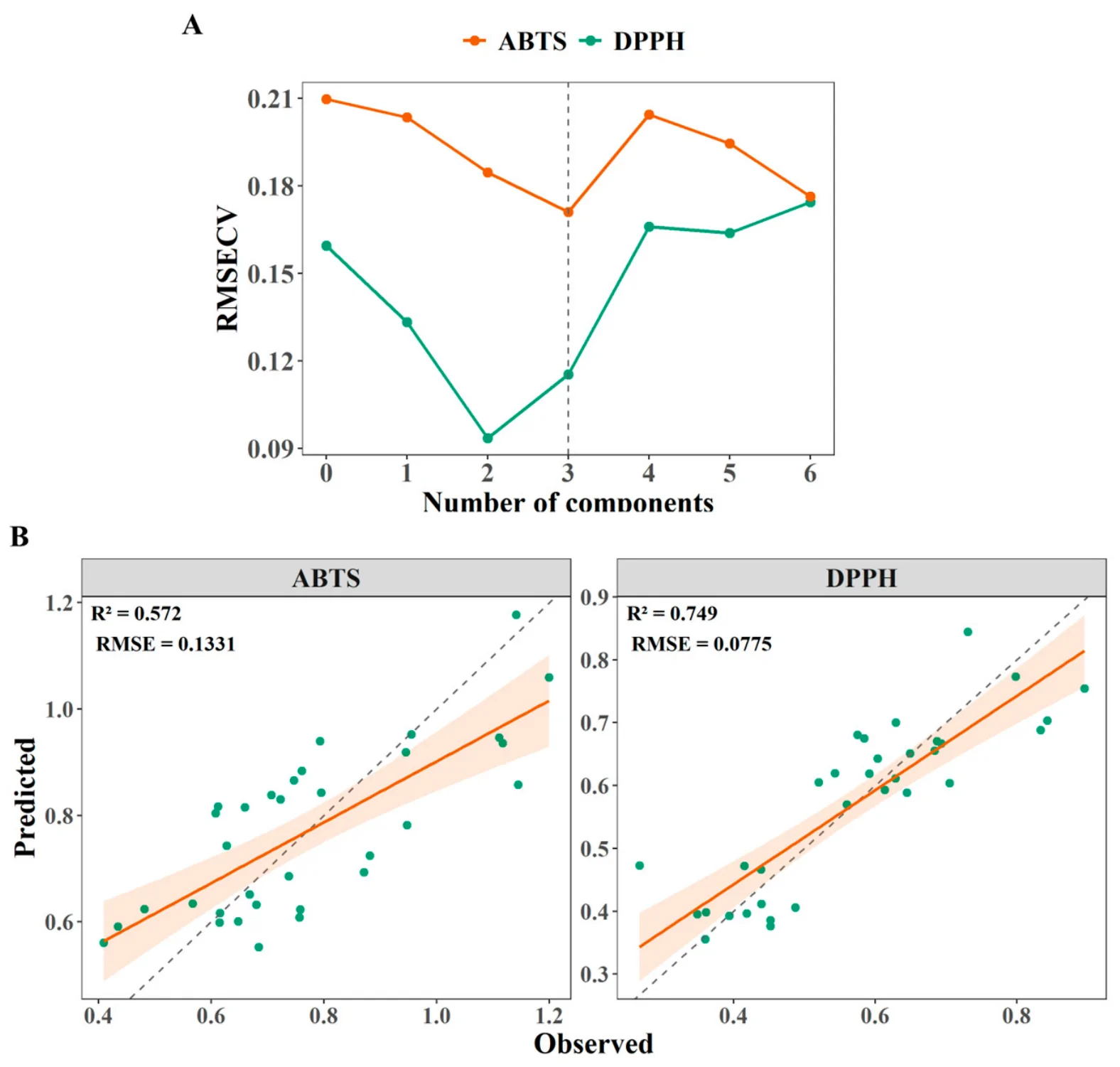
PLSR analysis of ginsenoside contents with DPPH and ABTS scavenging activities: (**A**) root mean square error of cross-validation (RMSECV) as a function of the number of latent variables; (**B**) scatter plot of measured vs. predicted values (The diagonal gray dashed lines represent the 1:1 line (y = x), indicating perfect agreement between measured and predicted values, while the orange solid lines represent the linear regression fit of the data).

The PLSR model used the six ginsenoside contents as independent variables and DPPH and ABTS scavenging rates as dependent variables for joint modeling, with the optimal number of latent variables being 3 (Figure 7A). The model showed better goodness-of-fit for DPPH (R^2^ = 0.749) than for ABTS (R^2^ = 0.572) (Figure 7B). The PLSR model was validated using a 10-fold cross-validation, the cross-validated R^2^ for DPPH was 0.665, while that for ABTS dropped sharply to 0.132, indicating that ginsenoside monomer contents can moderately predict DPPH activity but have extremely weak predictive ability for ABTS activity. This further supports the above inference: ginsenosides may mainly contribute to the scavenging of lipophilic radicals, while their scavenging effect on hydrophilic ABTS radicals is limited and probably rely on other residual components in the ginseng extracts such as polysaccharides and phenolic acids.

When modeling DPPH alone as the response variable, the training R^2^ (0.644) and cross-validation R^2^ (0.583) were both lower than those of joint modeling (Table 2), suggesting that although joint modeling did not improve the predictive ability for ABTS, it enhanced the stability and explanatory power of the DPPH model through multi-task learning. VIP ranking showed that the key active ginsenosides with the greatest contributions were Rb2, Rd, and Rc (VIP > 1, Table 3), consistent with the Pearson results. Notably, the regression coefficient for Rb2 was negative (Coef_DPPH = −0.0347, Table 3), contradicting the positive correlation coefficient from the Pearson results. We preferentially adopted the PLSR outcome because PLSR mitigates multicollinearity by projecting the original correlated variables onto a set of orthogonal latent components, whereas Pearson correlations could be inflated by collinearity.

## 3. Materials and Methods

### 3.1. Materials

Cultivated ginseng (*Panax ginseng* C. A. Mey.) and American ginseng (*Panax quinquefolius* L.) samples were collected from the ginseng cultivation base in Fusong County, Jilin Province. To uniformly compare the effects of growth years on ginsenoside contents, this study classified samples according to traditional commercial grades combined with growth years. The specific grading scheme is shown in Table 4. For each grade, three or six biological replicates were used, with each replicate representing an independent sample collected from separate cultivation plots to ensure representativeness. In addition, all samples were processed and analyzed in triplicate to assess method reproducibility. Six samples each of grades 1, 2, and 3, and three samples of grade 4 were collected for cultivated ginseng; three samples each of grades 1 to 4 were collected for American ginseng. All samples were dried, pulverized, and passed through a 60-mesh sieve, then sealed and stored for later use. Ginsenoside reference standards (Rb1, Rb2, Rc, Rd, Re, Rg1) were purchased from Chengdu Must Bio-Technology Co., Ltd. (Chengdu, China), with purities ≥ 98%. HPLC-grade acetonitrile and methanol were purchased from Kesite Technology Co., Ltd. (Chengdu, China), and ultrapure water was prepared using a Milli-Q system. DPPH (1,1-diphenyl-2-picrylhydrazyl),ABTS [2,2′-azino-bis(3-ethylbenzothiazoline-6-sulfonic acid)], and potassium persulfate were purchased from Solarbio (Beijing, China). Cellulase and pectinase (food grade) were purchased from Yuanye Co., Ltd. (Shanghai, China).

### 3.2. Extraction of Ginsenosides

Ultrasound-assisted dual-enzyme extraction was employed. Accurately weighed ginseng powder (1.0 g) was mixed with 100 U/g cellulase and 200 U/g pectinase, and 5 mL of 70% ethanol was added. The mixture was incubated in a water bath at 60 °C for 1 h for enzymatic hydrolysis, followed by ultrasonic extraction for 70 min (550 W). The extract was centrifuged (4000 rpm, 15 min), and the supernatant was filtered through a 0.22 μm membrane filter prior to HPLC analysis.

### 3.3. High-Performance Liquid Chromatography (HPLC)

The analysis of ginseng extracts was performed with a chromatographic column: Agilent ZORBAX SB-C18 (4.6 mm × 250 mm, 5 μm). Mobile phase A was 0.0088% phosphoric acid, and mobile phase B was acetonitrile. The gradient elution program was as follows: 0 min, 19% B; 0–35 min, 19% B; 35–55 min, 19% → 29% B; 55–70 min, 29% B; 70–100 min, 29% → 40% B; 100–101 min, 40% → 60% B; 101–111 min, 60% → 19% B. The flow rate was 1.0 mL/min, column temperature 35 °C, detection wavelength 203 nm, and injection volume 10 μL.

**Figure 8:**
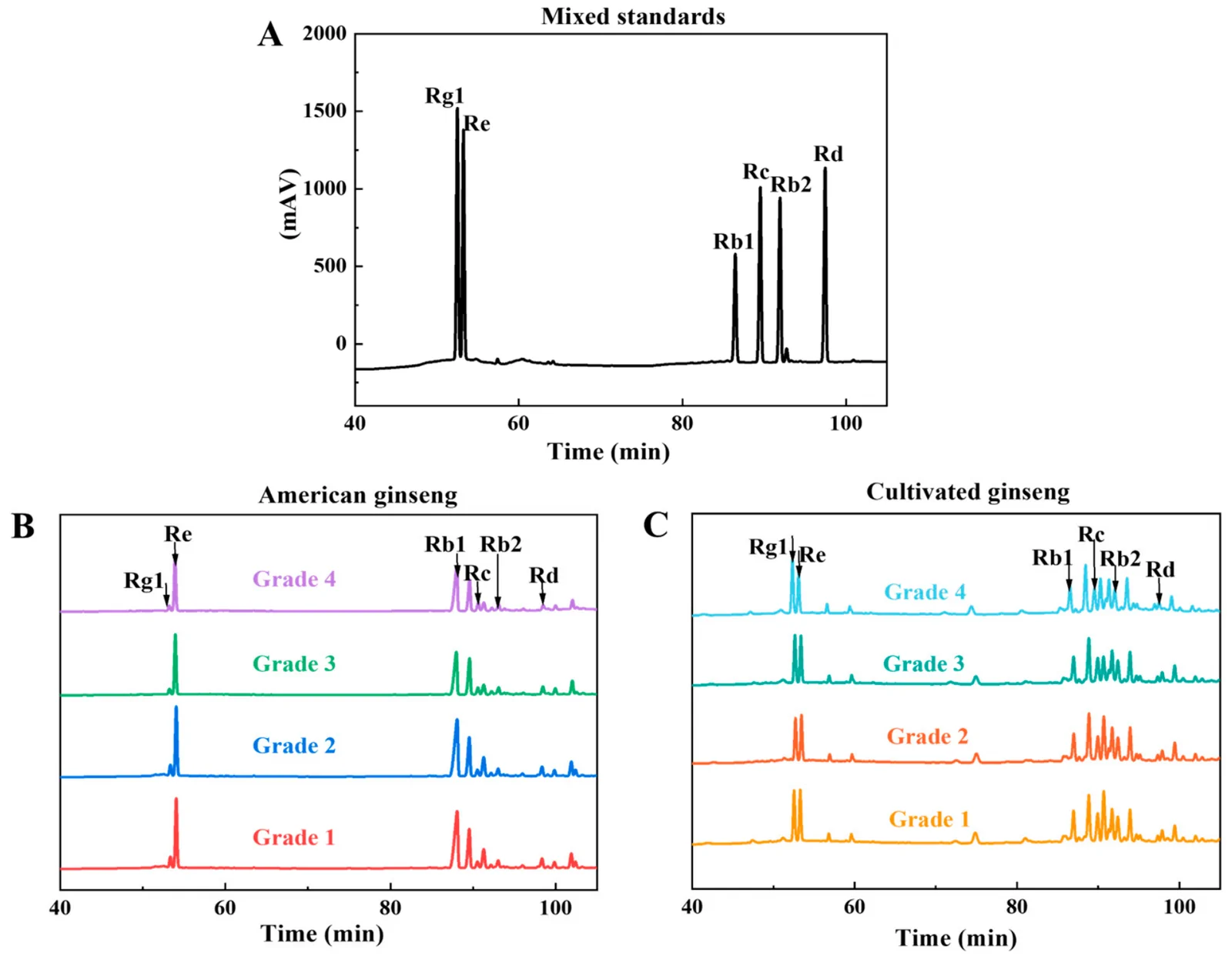
The representative HPLC chromatograms for (**A**) mixed standards, (**B**) American ginseng samples (grades 1–4), and (**C**) cultivated ginseng samples (grades 1–4).

Figure 8 showed representative HPLC chromatograms of mixed standards, cultivated ginseng, and American ginseng, demonstrating excellent separation of all six ginsenoside monomers under the optimized chromatographic conditions.

#### 3.3.1. Building of Standard Curve

Standard curve construction: Mixed reference solutions (concentrations shown in Table 5) were injected, peak areas were recorded, and regression equations were established with injection amount as the abscissa and peak area as the ordinate. The standard curve parameters (retention time, regression equation, linear range, and correlation coefficient) as well as the limits of detection and quantification (LOD and LOQ) for the six ginsenoside monomers are presented in Table 5.

#### 3.3.2. Method Validation

Precision: The test solution prepared from Grade 4 ginseng was accurately aspirated and injected continuously six times for HPLC analysis. The peak areas of ginsenosides Rb1, Rb2, Rc, Rd, Re, and Rg1 were recorded. The RSD values were 2.99%, 1.22%, 1.60%, 2.97%, 1.73%, and 1.53%, respectively, indicating good instrumental precision.

Repeatability: Six portions of Grade 4 ginseng powder (1.0114, 1.0117, 1.0301, 1.0201, 1.0214, and 1.0025 g) were accurately weighed. The test solutions were prepared according to the method described in Section 3.2 and analyzed by HPLC. The average contents of ginsenosides Rb1, Rb2, Rc, Rd, Re, and Rg1 were 0.588, 0.225, 0.366, 0.109, 0.269, and 0.538 mg/g, with RSD values of 2.42%, 1.07%, 0.73%, 1.01%, 2.75%, and 1.53%, respectively, demonstrating satisfactory repeatability of the analytical method.

Stability: The test solution prepared from Grade 4 ginseng was analyzed by HPLC at 0, 2, 4, 8, 12, and 24 h after preparation. The peak areas of ginsenosides Rb1, Rb2, Rc, Rd, Re, and Rg1 were recorded, and the corresponding RSD values were 2.62%, 2.15%, 1.43%, 2.74%, 1.86%, and 2.08%, respectively. The results revealed that the test solution remained stable within 24 h.

Accuracy (Spike recovery test): Approximately 1.0 g of ginseng powder with known analyte content was accurately weighed into six separate volumetric flasks. Appropriate volumes of mixed reference standard solution were spiked into each sample. The test solutions were prepared according to the method described in Section 3.2 and subjected to HPLC analysis. The peak areas of the six ginsenosides were recorded, and the spike recoveries were calculated using the external standard method. As shown in Table 6, the recoveries of all six ginsenosides ranged from 91.3% to 106.0%, with acceptable deviations, confirming the accuracy of the analytical method.

### 3.4. Determination of Antioxidant Activity

#### 3.4.1. DPPH Radical Scavenging Assay

A 100 μL aliquot of sample solution was mixed with 100 μL of 0.2 mmol/L DPPH ethanol solution and allowed to react in the dark for 30 min. The absorbance was measured at 517 nm ($A_{1}$). Absolute ethanol was used instead of DPPH as the background ($A_{2}$), and absolute ethanol instead of the sample as the control ($A_{0}$). The scavenging rate was calculated as:

$$\mathrm{Scavenging}\mathrm{rate}\left(\%\right)=\left(1-\frac{A_{1}-A_{2}}{A_{0}}\right)\times 100\%.$$

#### 3.4.2. ABTS Radical Scavenging Assay

ABTS working solution preparation: 7.4 mmol/L ABTS and 2.6 mmol/L potassium persulfate were mixed in equal volumes and stored in the dark for 12–16 h, then diluted with absolute ethanol to an absorbance of 0.70 ± 0.02 at 734 nm. A 100 μL aliquot of sample solution was mixed with 100 μL of ABTS^+^ working solution. After reaction for 6 min, the absorbance was measured at 734 nm. The scavenging rate calculation was the same as for DPPH.

### 3.5. Data Analysis

All experiments were performed in triplicate, and results are expressed as mean ± standard deviation. Statistical analyses were performed using R software (version 4.2.3) and related packages:

Principal component analysis (PCA): PCA was performed using the *prcomp* function with data scaled to unit variance (*scale = TRUE*). Score plots were generated using the *ggplot2* and *ggrepel* packages, with 95% confidence ellipses added using *stat_ellipse*.

Orthogonal partial least squares-discriminant analysis (OPLS-DA): OPLS-DA was performed using the *ropls* package (version 1.28.0). The model was constructed with one predictive component (*predI = 1*) and one orthogonal component (*orthoI = 1*), with data standardized by *scaleC = “standard”*. Model validity was assessed by 200 permutation tests (*permI = 200*) with 7-fold cross-validation (*crossvalI = 7*). A negative intercept of the permuted Q^2^ values indicated no overfitting. Variable importance in projection (VIP) scores > 1 were considered indicative of discriminant markers.

Hierarchical clustering analysis (HCA): HCA was performed using the *pheatmap* package (version 1.0.12) with Ward’s linkage method (*ward.D2*) and Euclidean distance as the dissimilarity measure. Data were scaled to unit variance before clustering.

Pearson correlation analysis: Pearson correlation coefficients were calculated using the *cor* function from the *psych* package (version 2.2.5), with significance testing at *p* < 0.05. Correlation matrices were visualized using the *corrplot* package (version 0.92).

Partial least squares regression (PLSR): PLSR was performed using the *pls* package (version 2.8.0) with the kernel PLS algorithm (*method = “kernelpls”*) and data scaling (scale = TRUE). The optimal number of latent variables was determined by 10-fold cross-validation (*segments = 10*) based on the minimum root mean square error of cross-validation (RMSECV). Model performance was evaluated by training R^2^ and cross-validated R^2^ (Q^2^). VIP scores were calculated to identify the most influential ginsenosides for antioxidant activity.

Two-way analysis of variance (ANOVA): Two-way ANOVA was performed using the *aov* function to evaluate the main effects of variety, grade, and their interaction on ginsenoside contents and antioxidant activities. A strictly balanced subset (2 varieties × 4 grades × 3 replicates, *n* = 24) was used to ensure orthogonality. When significant interactions were detected, Tukey’s honestly significant difference (HSD) post-hoc test was performed using the *multcomp* package (version 1.4-23) for multiple comparisons. The significance threshold was set at *p* < 0.05.

Data visualization: All figures were generated using the *ggplot2* package (version 3.4.2), with additional layout adjustments using *gridExtra* (version 2.3) and data reshaping using *reshape2* (version 1.4.4).

## 4. Conclusions

In this study, ultrasound-assisted dual-enzyme extraction was employed to extract ginsenosides from cultivated ginseng and American ginseng of different origins and grades. Combined with HPLC quantitative analysis and multivariate statistical methods, we systematically compared the content differences of six ginsenoside monomers (Rb1, Rb2, Rc, Rd, Re, Rg1) and their relationships with DPPH and ABTS free radical scavenging activities. The results demonstrated that variety is the primary factor affecting the ginsenoside profile. American ginseng contained significantly higher levels of PPD-type ginsenosides (Rb1, Rd, Rc), while cultivated ginseng was richer in the PPT-type ginsenoside Rg1. The effect of different grades (growth years) on ginsenoside contents did not show a monotonic trend, and the traditional view that “longer growth years yield superior components” should be treated with caution at the monomeric level. OPLS-DA demonstrated that cultivated ginseng and American ginseng could be clearly distinguished based on the contents of the six ginsenosides, with Rc, Rb1, and Rd identified as the key discriminatory ginsenosides (VIP > 1). HCA further revealed that Rd, Rb1, and Re exhibited similar accumulation patterns across samples, whereas Rg1 displayed a distinct profile separated from the others. Notably, Rg1 and Re, both belonging to the PPT type, showed a negative correlation in the loading plot, indicating that their abundance variations were oppositely regulated within the current sample pool, despite sharing the same aglycone type. Regarding antioxidant activity, American ginseng was slightly higher than cultivated ginseng, but the difference was not significant. Pearson correlation and PLSR models showed that DPPH scavenging rate was mainly positively correlated with Rb2, Rd, and Rc. The model exhibited moderate predictive ability for DPPH (cross-validation R^2^ = 0.665), while the correlation of ABTS scavenging rate with ginsenoside monomers was weak and predictive ability was extremely low (cross-validation R^2^ = 0.132), suggesting that there would be other underlying mechanism for ginseng extract to scavenge lipophilic radicals. PLSR joint modeling (with both DPPH and ABTS as response variables) showed superior fitting and predictive ability for DPPH compared to DPPH-alone modeling, indicating that joint modeling can improve model stability through multi-task learning. The VIP ranking identified Rb2, Rd, and Rc as the antioxidant-active ginsenosides with the greatest contributions. In summary, this study provides a multivariate statistical method based on six ginsenoside monomers for ginseng variety identification and preliminary antioxidant quality evaluation. These statistical associations, however, should be interpreted as hypothesis-generating rather than definitive mechanistic evidence; future studies incorporating LC-MS/MS verification, individual ginsenoside activity assays, and non-saponin components, as well as in vivo experiments, are warranted to further establish a comprehensive evaluation system for ginseng quality.

## Figures and Tables

**Table 1:** Two-way ANOVA results (F-values and *p*-values) for the effects of variety, grade, and their interaction on six ginsenoside monomers and antioxidant activities (DPPH and ABTS).

Response	Variety		Grade		Variety × Grade	
	F	*p*	F	*p*	F	*p*
Rb1	1287.65	<0.001	35.06	<0.001	30.46	<0.001
Rb2	34.34	<0.001	17.06	<0.001	15.46	<0.001
Rc	73.71	<0.001	10.19	<0.001	27.80	<0.001
Rd	620.97	<0.001	113.45	<0.001	54.50	<0.001
Re	260.60	<0.001	2.93	0.065	2.95	0.064
Rg1	164.21	<0.001	12.24	<0.001	43.01	<0.001
DPPH	3.51	0.079	11.24	<0.001	7.90	0.002
ABTS	0.23	0.639	7.25	0.003	2.03	0.151

**Table 2:** Comparison of performance between PLSR joint modeling and single-response modeling (training R^2^, RMSE, and cross-validation R^2^).

Model	Response Variable	Number of Components	R^2^ (Training)	RMSE (Training)	R^2^ (Cross-Validation)
DPPH + ABTS joint	DPPH	3	0.749	0.0775	0.665
DPPH + ABTS joint	ABTS	3	0.572	0.1331	0.132
DPPH alone	DPPH	1	0.644	0.0922	0.583

**Table 3:** Variable importance in projection (VIP) and regression coefficients for each ginsenoside monomer toward DPPH and ABTS scavenging activities.

Variable	VIP	Coef_DPPH	Coef_ABTS
Rb2	1.2701	−0.0347	−0.1502
Rd	1.1697	0.1163	0.2385
Rc	1.1322	0.1105	0.2007
Re	0.8806	−0.0603	−0.1772
Rb1	0.7801	0.0153	0.0079
Rg1	0.5939	−0.0345	−0.0688

**Table 4:** Information of ginseng samples (correspondence between grades and growth years in this experiment).

Variety	Grade	Corresponding Growth Year	Number of Samples
Cultivated ginseng	Grade 1	5 years	6
Cultivated ginseng	Grade 2	4 years	6
Cultivated ginseng	Grade 3	3 years	6
Cultivated ginseng	Grade 4	Below 3 years	3
American ginseng	Grade 1	5 years	3
American ginseng	Grade 2	4 years	3
American ginseng	Grade 3	3 years	3
American ginseng	Grade 4	Below 3 years	3

**Table 5:** Standard curve parameters for six ginsenoside monomers (retention time, regression equation, linear range, and correlation coefficient).

Ginsenoside	Regression Equation (mg/mL)	R^2^	Linear Range (mg/mL)	Retention Time (min)	LOD (mg/mL)	LOQ (mg/mL)
Rb1	Y = 2.0561x + 23,927.9	0.9995	0.25–2.5	86.845	0.05	0.15
Rb2	Y = 2.3210x + 96,108.5	0.9983	0.58–1.42	92.642	0.11	0.37
Rc	Y = 2.6784x + 19,045.3	0.9996	0.18–1.40	90.116	0.04	0.12
Rd	Y = 3.1448x + 7616.01	0.9998	0.10–0.70	97.546	0.02	0.06
Re	Y = 3.3021x + 127,686	0.9987	0.2–1.4	53.492	0.04	0.13
Rg1	Y = 2.9245x + 1295.33	0.9980	0.10–0.70	52.506	0.02	0.06

**Table 6:** Average spiked recovery of each ginsenoside.

Ginsenoside	Original Amount/mg	Spiked Amount/mg	Measured Amount/mg	Spike Recovery/%
Rb1	0.58	0.60	1.165	97.5 ± 1.33
Rb2	0.22	0.23	0.43	91.3 ± 2.30
RC	0.35	0.35	0.72	105.7 ± 1.70
Rd	0.10	0.10	0.22	95 ± 1.58
Re	0.25	0.25	0.54	106 ± 1.89
Rg1	0.53	0.55	1.07	98.18 ± 1.20

## Data Availability

The raw data supporting the conclusions of this article will be made available by the authors on request.
